# HILI destabilizes microtubules by suppressing phosphorylation and Gigaxonin-mediated degradation of TBCB

**DOI:** 10.1038/srep46376

**Published:** 2017-04-10

**Authors:** Hao Tan, Hua Liao, Lianfang Zhao, Yilu Lu, Siyuan Jiang, Dachang Tao, Yunqiang Liu, Yongxin Ma

**Affiliations:** 1 Department of Medical genetics, State Key Laboratory of Biotherapy, West china Hospital, Sichuan University, Chengdu, China

## Abstract

Human PIWIL2, aka HILI, is a member of PIWI protein family and overexpresses in various tumors. However, the underlying mechanisms of HILI in tumorigenesis remain largely unknown. TBCB has a critical role in regulating microtubule dynamics and is overexpressed in many cancers. Here we report that HILI inhibits Gigaxonin-mediated TBCB ubiquitination and degradation by interacting with TBCB, promoting the binding between HSP90 and TBCB, and suppressing the interaction between Gigaxonin and TBCB. Meanwhile, HILI can also reduce phosphorylation level of TBCB induced by PAK1. Our results showed that HILI suppresses microtubule polymerization and promotes cell proliferation, migration and invasion via TBCB for the first time, revealing a novel mechanism for HILI in tumorigenesis.

Microtubules are ubiquitous and versatile cytoskeletal structures connected with a wide variety of functions, such as cell division, cell motility and intracellular transport, all of which rely on microtubule dynamics^1^. Microtubule subunits are heterodimers composed of one α-tubulin polypeptide and one β-tubulin polypeptide. Correct incorporation of these subunits into the polymer requires a complex folding process and is facilitated by a subfamily of chaperones known as CCT/TriC/c-cpn and a set of tubulin specific cofactors (A to E)^2^,^3^.

TBCB, (Tubulin cofactor B), as its name suggests, is one of cofactor family (TBCA-TBCE) and plays a role in microtubule biosynthesis^4^,^5^,^6^,^7^,^8^,^9^,^10^,^11^,^12^,^13^,^14^. Overexpression of TBCB leads to microtubule depolymerization in HeLa cells. This function of TBCB is based on the ability of TBCB to form a binary complex with TBCE and greatly enhance the efficiency of TBCE to dissociate tubulin heterodimer^1^. The function of TBCB can be regulated by posttranslational modification. Previous research has shown that Gigaxonin interacts with TBCB and controls its degradation through the Ubiquitin-Proteasome pathway^7^. Previous research also showed that HSP90 (90-kDa heat-shock protein) is an abundant chaperone facilitating protein folding and stabilization. HSP90 can up-regulate TBCB expression^15^. In addition, recent research showed that growth factor-induced stimulation of p21-activated kinase 1 (PAK1) participates in regulating microtubule dynamics through phosphorylating TBCB on ser-65 and ser-128 during the microtububle regrowth phase^10^.

PIWIL2, aka HILI, is a member of PIWI subfamily containing PIWI and PAZ domains, plays crucial roles in self-renew of stem and germ cells, RNA silencing and translational regulation in different organisms during evolution and is ectopically expressed in different cancer cells^16^,^17^,^18^,^19^,^20^,^21^,^22^,^23^,^24^,^25^,^26^,^27^,^28^,^29^,^30^,^31^,^32^. Our previous researches have shown that HILI regulates microfilaments and intermediate filaments of tumor cells^29^,^30^,^31^,^32^, these findings prompt us to shift our attention to the roles of HILI in microtubule dynamics of tumor cells.

Here we present that HILI suppresses microtubule polymerization and promotes cell proliferation, migration and invasion via TBCB for the first time. Our current study reveals that HILI inhibits TBCB ubiquitination and degradation and reduces phosphorylation level of TBCB induced by PAK1, revealing a novel mechanism for HILI in tumorigenesis.

## Results

### HILI suppresses microtubule polymerization in a TBCB-dependent manner

**Figure 1:**
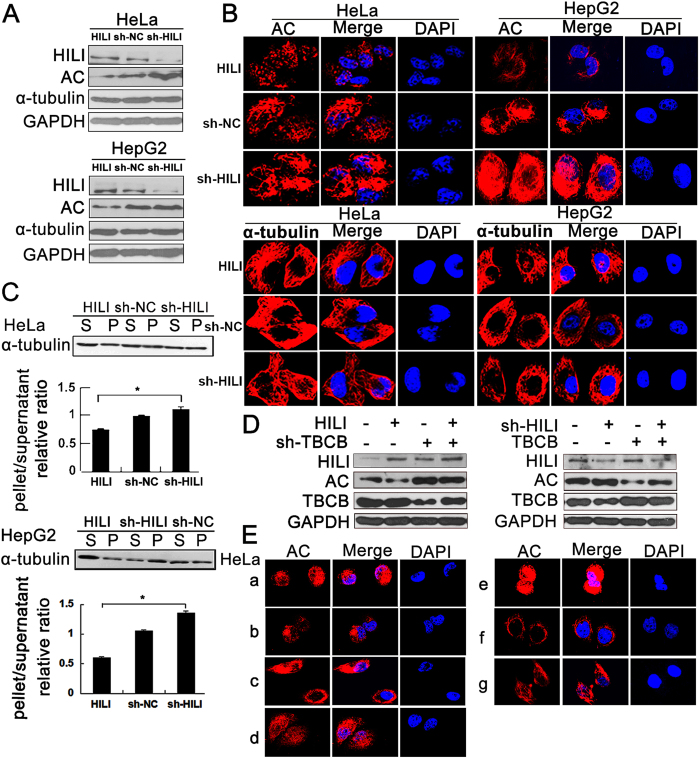
HILI suppresses microtubule polymerization in a TBCB-dependent manner. (**A**) HILI down**-**regulated acetylation level of α-tubulin and had no significant effect on α-tubulin expression at protein level in HeLa and HepG2 cells. HeLa and HepG2 cells were transfected with MYC-HILI, sh-NC, or sh-HILI vector. After 48 h, cell lysates were prepared for Western blotting with AC-α-tubulin and α-tubulin antibody (AC, AC-α-tubulin). (**B**) Immunofluorescent staining of AC-α-tubulin and α-tubulin in transfected cells. (**C**) *In vivo* tubulin polymerization assays in HeLa and HepG2 cells. Supernatant (S) and pellet (P) fractions of cell lysates were analyzed with anti-α-tubulin, data were presented as mean ± sd. (*P < 0.05). (**D**) Knockdown of TBCB recovered acetylation level of α-tubulin decreased by HILI overexpression, overexpression of TBCB inhibited the increase of acetylation level of α-tubulin induced by HILI knockdown. (**E**) Immunofluorescence assays showed that HILI down-regulated acetylation level of α-tubulin in a TBCB-dependent manner (a, NC; b, HILI; c, sh-TBCB; d, HILI/sh-TBCB; e, sh-HILI; f, TBCB; g, sh-HILI/TBCB; AC, AC-α-tubulin).

Acetylated α-tubulin can only be detected in polymerized microtubules^33^,^34^,^35^. To prove whether there is a relationship between HILI and microtubule, we detected the change of acetylation level of α-tubulin and α-tubulin expression altered by HILI. Western blotting analyses showed that HILI overexpression decreased acetylation level of α-tubulin and HILI knockdown increased acetylation level of α-tubulin, but α-tubulin expression was not significantly changed in HeLa and HepG2 cells (Fig. 1A). Laser confocal microscopy (LSCM) was also introduced to detect the acetylation level of α-tubulin, showing that up-regulation of HILI decreased acetylation level of α-tubulin and down-regulation of HILI increased acetylation level of α-tubulin in HeLa and HepG2 cells, but fluorescence intensity of α-tubulin had no significant change (Fig. 1B). To further prove that HILI inhibits microtubule polymerization, a tubulin polymerization *in vivo* assay was performed, showing that HILI overexpression significantly decreased polymerized α-tubulin and HILI knockdown significantly increased polymerized α-tubulin in HeLa and HepG2 cells (Fig. 1C). We further studied how HILI inhibited microtubule polymerization.When TBCB was overexpressed simultaneously, HILI knockdown can no longer increase the acetylation level of α-tubulin and *vice versa* (Fig. 1D). Similar results were also observed in immunofluorescence experiments (Fig. 1E). These results suggested that HILI inhibits microtubule polymerization in a TBCB-dependent manner.

### HILI interacts with TBCB

**Figure 2:**
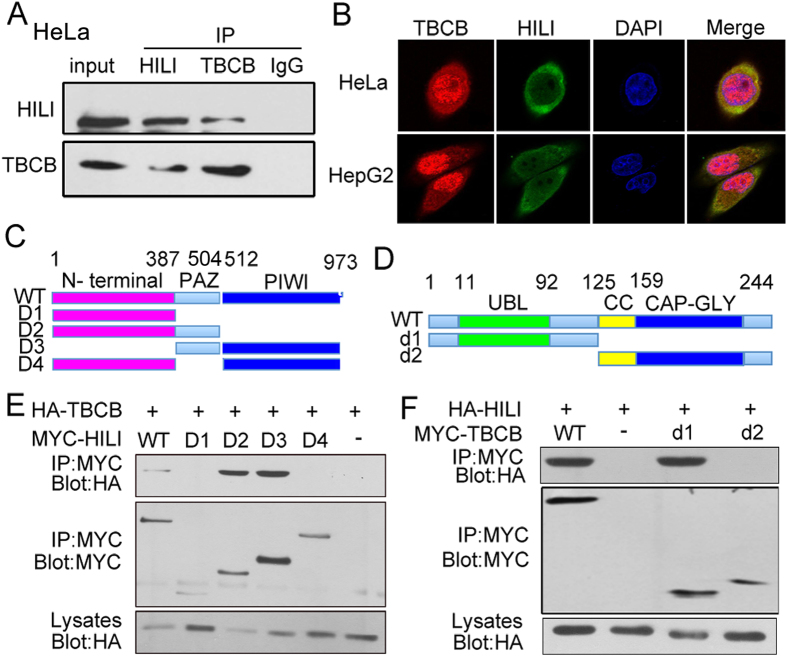
HILI interacts with TBCB. (**A**) Endogenous interaction between HILI and TBCB. (**B**) Immunofluorescence assays showed that HILI and TBCB were mainly overlapped in cytoplasm. (**C**) Schematic of HILI deletion mutants. (**D**) Schematic of TBCB deletion mutants. (**E**) Interaction between TBCB and different MYC-tagged HILI mutants. (**F**) Interaction between HILI and different MYC-tagged TBCB mutants.

We have proved that HILI suppresses microtubule polymerization in a TBCB-dependent manner, so we next confirmed whether HILI can interact with TBCB in tumor cells. Subsequently coimmunoprecipitation assays were performed, showing that endogenous HILI and TBCB can interact with each other in HeLa cells (Fig. 2A), and then immunofluorescence assays showed that endogenous HILI and TBCB were mainly overlapped in cytoplasm in HeLa and HepG2 cells (Fig. 2B). To further identify the functional domains involved in the interaction between HILI and TBCB, we constructed their mutants respectively (Fig. 2C,D). Results showed that mutants of HILI lacking the PAZ domain such as D1 or D4 failed to interact with TBCB (Fig. 2E). These results suggested that PAZ domain plays an important role in the binding between HILI and TBCB. We next mapped the HILI binding site in TBCB. TBCB mutants lacking the UBL domain such as d2 failed to interact with HILI (Fig. 2F). These results suggested that the HILI binding site in TBCB is localized in the UBL functional domain.

### HILI up-regulates TBCB expression through inhibiting Gigaxonin-mediated ubiquitination and degradation of TBCB

**Figure 3:**
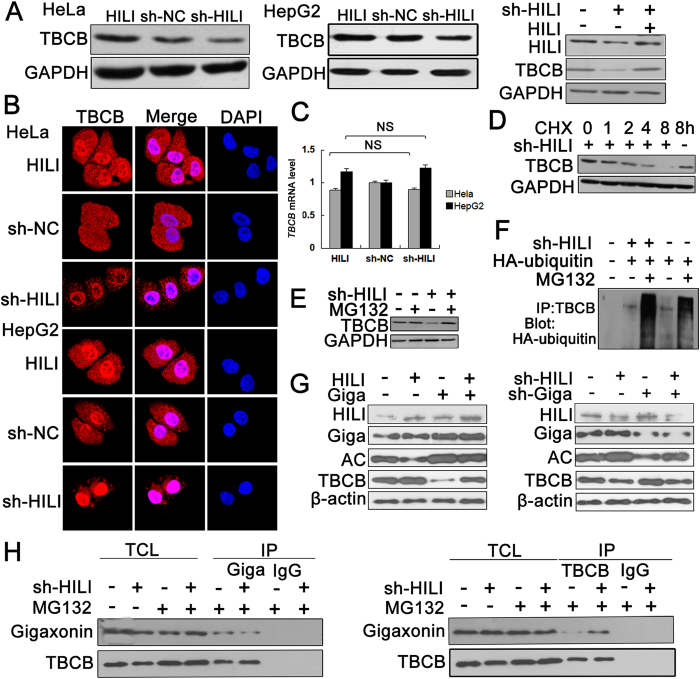
HILI promotes TBCB expression through blocking Gigaxonin mediated ubiquitination and degradation of TBCB. (**A**) HILI up-regulated TBCB expression at protein level in HeLa and HepG2 cells. (**B**) Immunofluorescent staining of TBCB in transfected HeLa and HepG2 cells with TBCB antibodies. (**C**) Real-time PCR assays showed that HILI had no significant effect on TBCB mRNA level, data were presented as mean ± sd (NS, P > 0.05). (**D**) HILI knockdown accelerated TBCB degradation. (**E**) MG132 recovered TBCB expression reduced by HILI knockdown. (**F**) Western blotting results showed that the level of poly-ubiquitination of TBCB increased in HILI knockdown cells compared with control cells. HeLa cells were transfected with HA-ubiquitin vector, followed with treatment of MG132 for 6 h and precipitated with TBCB antibodies for Western blotting analysis. (**G**) HILI inhibited Gigaxonin-mediated degradation of TBCB (Giga, Gigaxonin; AC, AC-α-tubulin). (**H**) HILI knockdown promoted the interaction between Gigaxonin and TBCB.

Based on above findings, we next examined whether HILI can affect TBCB expression. Western blotting assays showed that overexpression of HILI increased TBCB expression and knockdown of HILI decreased TBCB expression. Meanwhile, the decrease of TBCB expression regulated by HILI knockdown can be restored by overexpression of HILI (Fig. 3A). Immunofluorescence experiments also showed that HILI knockdown decreased TBCB fluorescence intensity and HILI overexpression increased TBCB fluorescence intensity in HeLa and HepG2 cells (Fig. 3B). The change of TBCB protein level may be due to protein degradation and/or change at mRNA level. We first performed real-time PCR assays and then no significant difference was observed after altering HILI expression (Fig. 3C). In cells treated with cycloheximide (CHX) to inhibit protein synthesis, HILI knockdown increased degradation of TBCB (Fig. 3D). In cells treated with proteasome inhibitor MG132, the down-regulation of TBCB induced by HILI knockdown was rescued (Fig. 3E). Ubiquitination assay showed that the level of poly-ubiquitination of TBCB increased in HILI knockdown cells compared with control cells (Fig. 3F).

Previous research has shown that Gigaxonin interacts with TBCB and controls its degradation through the ubiquitin-proteasome pathway^7^. So we detected what roles HILI plays in TBCB ubiquitination and degradation. Western blotting assays showed that HILI inhibited Gigaxonin-mediated degradation of TBCB (Fig. 3G). Coimmunoprecipitation assays showed that HILI knockdown promoted the binding between Gigaxonin and TBCB (Fig. 3H). Taken together, HILI interacts with TBCB to inhibit the interaction between Gigaxonin and TBCB thus inhibiting Gigaxonin-mediated ubiquitination and degradation of TBCB.

### HILI inhibits Gigaxonin-mediated TBCB ubiquitination and degradation in the HSP90-dependent manner

**Figure 4:**
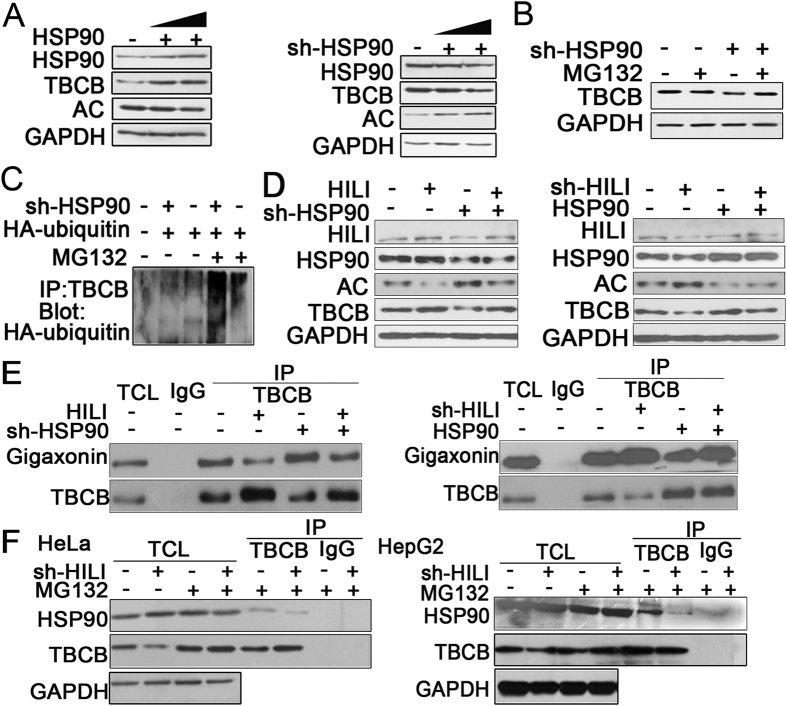
HILI inhibits Gigaxonin**-**mediated ubiquitination and degradation of TBCB in the HSP90-dependent manner. (**A**) HSP90 up-regulated TBCB expression at protein level. (**B**) MG132 recovered TBCB expression reduced by HSP90 knockdown. (**C**) HeLa cells were transfected with HA-ubiquitin vector, followed with treatment of MG132 for 6 h and precipitated with TBCB antibodies for Western blotting analysis. (**D**) HILI inhibited Gigaxonin**-**mediated ubiquitination and degradation of TBCB in the HSP90-dependent manner. (**E**) HILI regulated the interaction between Gigaxonin and TBCB in the HSP90-dependent manner. (**F**) HILI promoted the interaction between HSP90 and TBCB.

The previous research showed that HSP90 acts as chaperone of TBCB and stabilize TBCB^15^. Our previous research also showed that HILI interacts with HSP90 to prevent formation of HSP90-TβR complex and improve ubiquitination and degradation of TGF-β receptors. So we studied whether HILI has an effect on TBCB expression in the HSP90-dependent manner. The results showed that TBCB protein level was increased and acetylation level of α-tubulin was reduced in HSP90 overexpressed cells and *vice versa* (Fig. 4A). Meanwhile, the decrease of TBCB expression induced by knockdown of HSP90 was restored upon MG132 treatment (Fig. 4B) and HSP90 konckdown increased the level of poly-ubiquitination of TBCB (Fig. 4C). Our further experiments showed that HSP90 knockdown can restore the increase of TBCB expression by HILI, and overexpression of HSP90 rescued the decrease of TBCB expression induced by HILI knockdown (Fig. 4D). Immunoprecipitation experiments showed that HSP90 knockdown restored the interaction between Gigaxonin and TBCB decreased by HILI overexpression and HSP90 overexpression decreased the interaction between Gigaxonin and TBCB increased by knockdown of HILI (Fig. 4E). Meanwhile, HILI knockdown decreased the interaction between HSP90 and TBCB (Fig. 4F). Taken together, HILI inhibits Gigaxonin-mediated TBCB ubiquitination and degradation by promoting the interaction between HSP90 and TBCB and inhibiting the binding between Gigaxonin and TBCB.

### HILI, HSP90 and TBCB form a protein complex

**Figure 5:**
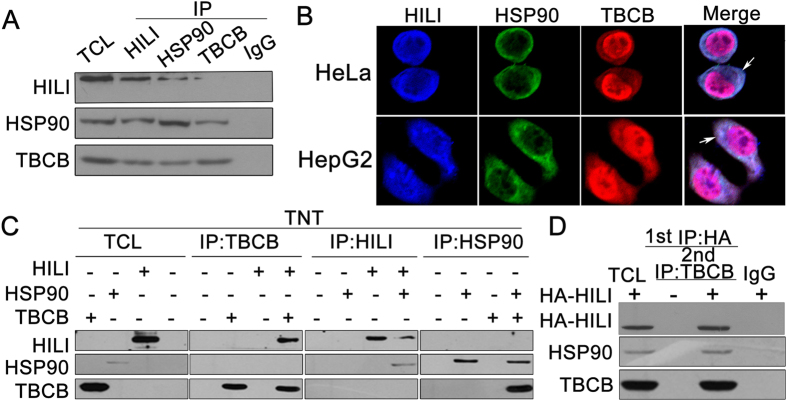
HILI, HSP90 and TBCB form a protein complex. (**A**) Co-immunoprecipitation assays showed the interaction of HILI, HSP90 and TBCB. (**B**) Colocalization of HILI, HSP90 and TBCB (white arrows indicates the overlapped areas). (**C**) Each of HILI, HSP90 and TBCB can directly interact with each other *in vitro* by TNT^®^ Quick Coupled Transcription/Translation Systems. (**D**) A two-step immunoprecipitation assay showed the existence of a complex comprising HILI, TBCB and HSP90.

Our previous research has proved that HILI can interact with HSP90^30^. Based on our above finding that HILI interacts with TBCB, we hypothesized that HILI, HSP90 and TBCB may form a protein complex. Coimmunoprecipitation assays showed that each of these three proteins can interact with each other (Fig. 5A). Immunofluorescence assays also showed that HILI, HSP90 and TBCB were mainly overlapped in cytoplasm (Fig. 5B). Meanwhile, each of HILI, HSP90 and TBCB can directly interact with each other *in vitro* by TNT^®^ Quick coupled Transcription/Translation Systems (Fig. 5C). A two-step immunoprecipitation assay further confirmed the existence of a complex comprised of HILI, TBCB and HSP90 (Fig. 5D).

### HILI inhibits PAK1-induced phosphorylation of TBCB

**Figure 6:**
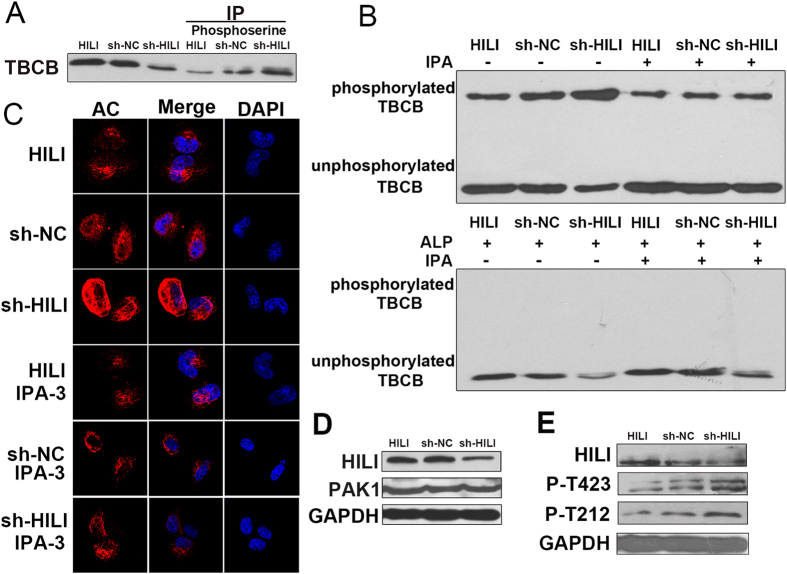
HILI inhibits PAK1**-**induced phosphorylation of TBCB. (**A**) Immunoprecipitation assay showed that HILI decreased phosphorylation level of TBCB. (**B**) HILI reduced phosphorylation level of TBCB. When PAK1 inhibitor IPA-3 was added, HILI no longer decreased the phosphorylation level of TBCB. The bands of phosphorylated TBCB was identified by ALP (alkalinephosphatase) treatment. (**C**) Immunofluorescence assays showed that PAK1 inhibitor IPA-3 inhibited the regulation of α-tubulin acetylation level by HILI. (**D)** HILI had no significant effect on PAK1 expression. (**E**) HILI overexpression reduced phosphorylation level of PAK1 on Thr-423 and Thr-212.

Recent research has shown that the posttranslational phosphorylation of TBCB regulated by PAK1 plays an important role in microtubule dynamics^10^, and we have proved that HILI inhibits microtubule polymerization via TBCB. So we propose a hypothesis that HILI may have an effect on PAK1 which can induce TBCB phosphorylation and microtubule dynamics. To confirm this, immunoprecipitation assay was performed, showing that HILI overexpression decreased phosphorylation level of TBCB (Fig. 6A). Phos-tag™ Acrylamide SDS-PAGE gel was also employed to show bands of phosphorylated TBCB. When cells were treated with PAK1 inhibitor IPA-3, HILI overexpression no longer decreased phosphorylation level of TBCB (Fig. 6B), suggesting that HILI inhibits PAK1-induced TBCB phosphorylation. Immunofluorescence assays showed that HILI inhibited the increase of α-tubulin acetylation level induced by PAK1 (Fig. 6C). Meanwhile, HILI had no significant effect on PAK1 expression and HILI overexpression reduced phosphorylation level of PAK1 on Thr-423 and Thr-212 (Fig. 6D,E). These results suggested that HILI decreases phosphorylation level of PAK1, reduces phosphorylation level of TBCB and inhibits microtubule polymerization.

### HILI promotes cell proliferation, migration and invasion via TBCB

**Figure 7:**
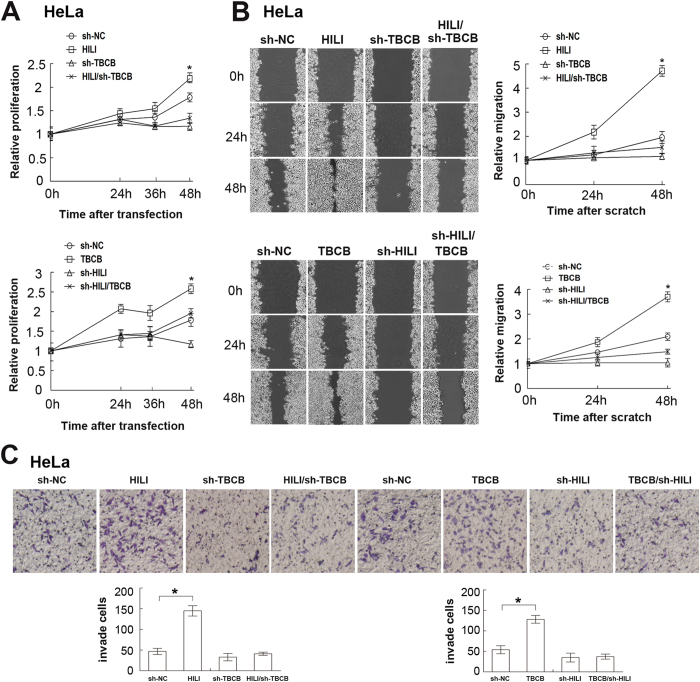
HILI promotes cell proliferation, migration and invasion via TBCB. (**A**) Cell Counting Kit-8 assays showed that TBCB knockdown inhibited cell proliferation promoted by HILI overexpression and TBCB overexpression recovered cell proliferation inhibited by HILI knockdown. (**B**) Scratch wound assays showed that HILI promoted cell migration via TBCB. (**C**) HILI promoted tumor cell invasion through TBCB. Hela cells were incubated with filter chambers coated with Matrigel and cultured for 24 h. Cells on the upper side of the membrane were then removed and underside cells were fixed, stained with Wright Giemsa. Data were presented as mean ± sd. (*P < 0.05).

Recent research has shown that cancer cells depend on their cytoskeleton, including actin, microtubules and intermediate filaments, to proliferate, invade and metastasize^4^. Our previous researches showed that HILI can promote cell proliferation^29^,^32^. Based on our findings above, we studied whether TBCB is implicated in HILI-regulated tumor cell proliferation, migration and invasion. First, cell counting kit-8 (CCK-8) assays were performed, showing that TBCB knockdown suppresses cell proliferation promoted by HILI overexpression and *vice versa* (Fig. 7A). Then we performed cell scratch wound assay and cell invasion experiment, and the results showed that HILI and TBCB overexpression can promote migration and invasion baffled by HILI and TBCB knockdown, and when HILI and TBCB were knockdowned simultaneously, the situation of cell migration was impeded (Fig. 7B,C). Taken together, these findings suggested that HILI promotes cell proliferation, migration and invasion via TBCB.

## Discussion

Cytoskeleton, composed of microtubule, intermediate filament and microfilament, plays important roles in cell division, cell differentiation, cell apoptosis and cell cancerization^36^,^37^,^38^. Our previous researches have shown that HILI plays significant roles in regulating intermediate filament and microfilament in tumor cell^29^,^32^. In the present study, our results showed that HILI can also inhibit microtubule polymerization in a TBCB-dependent manner (Fig. 1).

TBCB, one member of cofactor family, is of great importance in proper folding α/β-tubulin to form heterodimers which are then polymerized into microtubules^1^. TBCB is a microtubule-destabilizing factor, and accumulation of TBCB in cells may be a causative factor responsible for microtubule pathology in human GAN (giant axonal neuropathy)^7^. TBCB can be regulated by posttranslational modification, including ubiquitination and phosphorylation^7^,^10^. Our current study showed that HILI can interact with TBCB (Fig. 2) and inhibit TBCB degradation (Fig. 3A–F). Gigaxonin can participate in TBCB ubiquitination and degradation as E3 ubiquitin ligase^7^. We further showed that HILI can inhibit the interaction between Gigaxonin and TBCB thus inhibiting Gigaxonin-mediated TBCB ubiquitination and degradation (Fig. 3G,H).

Our results showed that HSP90 can upregulate TBCB (Fig. 4A), and this result is consistent with previous study^15^. As shown in Fig. 4C, HSP90 can inhibit ubiquitination of TBCB. Our results showed that HILI can promote the interaction between HSP90 and TBCB, and HSP90 overexpression can inhibit the interaction between Gigaxonin and TBCB led to TBCB ubiquitination and degradation. This suggests that HSP90 may be a chaperone protein of TBCB (Fig. 4). Coimmunoprecipitation assays, immunofluorescence assay, transcription and translation assay *in vitro* and two-step immunoprecipitation assay further showed that HILI, HSP90 and TBCB can form a protein complex (Fig. 5). This prompts that HILI can promote the interaction between HSP90 and TBCB, inhibit the interaction between Gigaxonin and TBCB, and then suppress TBCB ubiquitination and degradation.

In addition, TBCB can be phosphorylated by the serine/threonine p21-Activated Kinase 1 (PAK1), and phosphorylation of TBCB can enhance polymerization of new microtubules^10^. Our research showed that HILI inhibits PAK1-induced phosphorylation of TBCB thus inhibiting microtubule polymerization (Fig. 6), this prompts us that HILI may affect cell proliferation by influencing TBCB phosphorylation.

Cytoplasmic microtubules depolymerize and restructure into spindle in the early stage of the cell division. The dynamics of microtubule assembly make it unique in the cell division^39^. Destruction of microtubules can result in inhibition of protrusive lamellipodial activity^40^,^41^, disrupting microtubules also led to Rho activation thus promoting actin cytoskeleton retraction of cell rear^42^,^43^, so microtubule plays important role in cell migration. Our previous researches have shown that HILI promotes tumor cells proliferation^29^,^30^,^31^,^32^,^33^. Here, we showed that HILI promotes tumor cell proliferation, migration and invasion via TBCB (Fig. 7).

**Figure 8:**
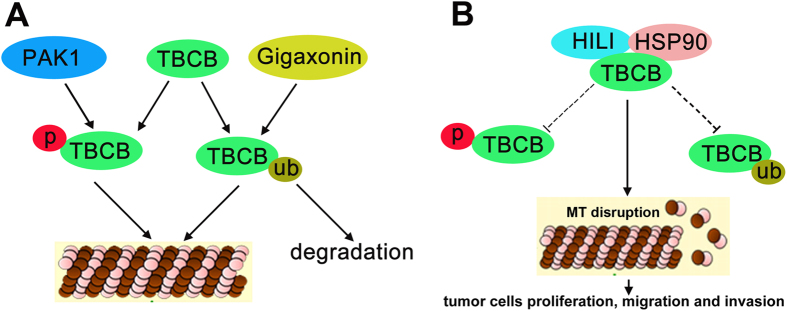
Model of how HILI regulates microtubule dynamics via TBCB.

In summary, our results showed that HILI regulates microtubule dynamics through two path ways. First, HILI interacts with TBCB and promotes the interaction between HSP90 and TBCB to inhibit the interaction between Gigaxonin and TBCB and suppress Gigaxonin mediated ubiquitination and degradation of TBCB. Second, HILI inhibits TBCB phosphorylation induced by PAK1. The up-regulation of TBCB expression and down-regulation of TBCB phosphorylation level induced by HILI overexpression suppress microtubule polymerization. Thus HILI can promote tumor cells proliferation, migration and invasion via TBCB (Fig. 8). Considering that microtubule dynamics contributes to a variety of significant bioprocess, our research provides a new perspective on the role of HILI in tumor cell proliferation, migration and invasion and extends the function of the PIWI protein in tumorigenesis.

## Methods

### Plasmid construction, shRNA and antibodies

cDNAs encoding MYC-tagged HILI, HA-tagged HILI, HA-tagged TBCB, HA-tagged Gigaxonin, and HA-tagged HSP90 were synthesized and inserted into pcDNA3.1(+) expression vector (Invitrogen). Meanwhile, we constructed a series of MYC-tagged HILI mutants (described in Fig. 1C) and MYC -tagged TBCB mutants (described in Fig. 1D) by segment and fusion PCR, and non-specific random shRNA control (sh-NC) was purchased from Gene Pharma (Shanghai, China).shRNA for HILI, TBCB and HSP90 were synthesized and cloned into pGPU6/GFP/Neo. The target sequences of these shRNA were as follows:

HILI shRNA (sh-HILI): 5′- CUAUGA GAUUCCUCAACUACAGAAG-3′

TBCB shRNA (sh-TBCB): 5′- AATGGGAAACGCTACTTCGAA-3′

HSP90 shRNA (sh-HSP90): 5′- GGA AAG AGC UGC AUA UUA ATT-3′

The following polyclonal antibodies (pAbs) and monoclonal antibodies (mAbs)

were used: anti-HILI (pAb, sc-67303), anti-TBCB (mAb, sc-377139), anti-HSP90 (pAb, sc-1055), anti-Gigaxionin (mAb, sc-376173), anti-MYC (pAb, sc-789), anti-HA (pAb, sc-805), anti-PAK1 (T-212, pAb, sc-101772) and anti-PAK1(T-423, pAb, sc-12925) were purchased from Santa Cruz Biotechnology (Santa Cruz, USA); anti-Ac-α-tubulin (mAb, 5335) was purchased from Cell Signaling Technology (Boston, USA); anti-Phosphoserine (pAb, ab9332) and anti-GAPDH (mAb, ab181602) were purchased from abcam (Cambridge, MA); anti-α-tubulin (mAb, AT819) was purchased from Beyotime Biotechnology (Shanghai, China).

### Cell culture, Transfection and Treatment

HeLa and HepG2 cells were maintained in our laboratory as previously described^27^,^30^. They were cultured in DMEM with 10% FBS and transfected by using jetPRIME (#114-15, SA, France) according to manufacturers’ protocols. All transfections were performed in 6-well plates. Forty-eight hours after transfection, cycloheximide (CHX) was added to a final concentration of 6 μg/ml for 0–8 h. Where specified, cells were treated with the proteasome inhibitor MG132 at a final concentration of 10 μM. Cells were harvested and analyzed by Western blotting using appropriate antibodies. All following experiments were repeated at least three times unless stated otherwise.

### Coimmunoprecipitation and Western blotting

For coimmunoprecipitation and western blotting, cells were lysed after transfecting with designated plasmids in universal protein extraction buffers (#PP1801, Bioteke, China) containing protease inhibitor (#04693116001, Roche, Switzerland). Extracted proteins were immunoprecipitated with special antibody and protein A + G agarose beads (#P2012, Beyotime, China). Bound proteins were separated using SDS-PAGE, transferred to polyvinylidene difluoride membranes (#IPVH00010, Millipore, USA) and detected with specific appropriate primary antibodies and horseradish peroxidase-conjugated secondary antibodies. Specific proteins were visualized using an enhanced chemiluminescence (ECL) Western blotting detection system (#WBKLS0100, Millipore, USA).

### Immunofluorescence staining

Cells were fixed with 4% paraformaldehyde in PBS for 10 min and permeabilized with 0.5% Triton X-100 for 6 min, blocked with 1% BSA for 30 min, incubated overnight at 4 °C with primary antibody, and finally incubated with Alexa Fluor^®^ 488 (#A-11055, Thermo Fisher Scientific, USA), Alexa Fluor^®^ 555 (#A-31570, Thermo Fisher Scientific, USA), Alexa Fluor^®^ 350 (#A10039, Thermo Fisher Scientific, USA) for 1 hour at room temperature. Each step was followed by two 5 min washes in PBS. For nuclear stain, prepared specimens were counterstained with DAPI (1‰) for 2 min. Fluorescent images were obtained with a confocal microscope (Olympus, Japan).

### Quantitative real-time PCR

Total RNA was prepared using TRIzol (#15596-026, Invitrogen, USA) from HeLa and HepG2 cells transfected with MYC-HILI, sh-HILI or sh-NC. Quantitative PCR was performed in a CFX96 Touch real-time PCR detection system (Bio-Rad, USA) with a denaturation step at 94 °C for 10 min, followed by 45 cycles of denaturation at 94 °C for 20 s, annealing at 60 °C for 30 s, and extension at 72 °C for 40 s using the Kapa SYBR (#KK4650, Kapa. Boston, USA).

### *In vitro* binding assay

For protein interaction analysis, *in vitro* protein binding assays were performed using TNT^®^ Quick Coupled *in vitro* transcription/translation system (#REFL1171, Promega, USA) according to the manufacturer’s instructions. The reactions were carried out in 25 μl volumes by adding 1 μg of plasmid DNA and 1 μl unlabeled methionine to the TnT mixture. We incubated the reaction mixture at 30 °C for 90 min and then 2 μl of each production was used to detect the protein expression. Subsequently, 20 μl aliquots of each protein were mixed together in 200 μl binding buffer (20 mM Tris-HCl, pH 7.5; 150 mM NaCl; 1 mM dithiothreitol; 0.1% Tween 20) with protease inhibitors and incubated on a rotating platform at 4 °C for 3 h. Interactions were detected by coimmunoprecipitation and western blotting using specific antibodies.

### Cell counting kit-8 (CCK-8) assays

Cell proliferation was analyzed using WST-8 Cell Counting Kit-8 (#C0038, Beyotime, China). Cells were seeded in 96-well plates with 100 μl of DMEM medium containing 10% FBS and cultured in a humidified incubator (at 37 °C, 5% CO_2_). Add 10 μl of the CCK-8 solution into each well of the plates at indicated time after transfection, and then incubate the plates for 2 hours in the incubator (at 37 °C, 5% CO_2_). Measure the absorbance at 450 nm using a microplate reader (BioTek, Vermont, USA). The relative proliferation ratio was presented as fold change that was calculated by absorbance and normalized by control to an arbitrary value of one.

#### Cell migration and invasion assays

Transfected cells were cultured in serum-free medium for 16 h. We created linear wounds with a pipette tip and observed after 24 h and 48 h. Images were captured using a fluorescence inverse microscope (Olympus, Japan). For cell invasion assays, cells were cultured in serum-free medium for 24 h, plated into the top transwell filter chambers coated with Matrigel (#354230, Corning, USA) and incubated for 24 h at 37 °C with 5% CO_2_. Migratory cells were fixed in methanol for 15 min, and stained with Wright-Giemsa (Jiancheng Biotech, Nanjing, China).

### Statistical analysis

Experiments were repeated three times, and all data were presented as the mean ± sd using GraphPad Prism 5.0 software. Migration and invasion results were analyzed with Image-Pro Plus software. Statistical analyses were performed using SPSS version 17.0. Differences between experimental groups were determined using Student’s test. Values of P < 0.05 were considered as significant.

## Additional Information

**How to cite this article:** Tan, H. *et al*. HILI destabilizes microtubules by suppressing phosphorylation and Gigaxonin-mediated degradation of TBCB. *Sci. Rep.* **7**, 46376; doi: 10.1038/srep46376 (2017).

**Publisher's note:** Springer Nature remains neutral with regard to jurisdictional claims in published maps and institutional affiliations.

## References

[b1] Kortazar D. . Role of cofactors B (TBCB) and E (TBCE) in tubulin heterodimer dissociation. Exp. Cell Res. 313, 425–436 (2007). 17184771 10.1016/j.yexcr.2006.09.002

[b2] Lopez-Fanarraga M., Avila M. J., Guasch A., Coll M. & Zabala J. C. Postchaperonin tubulin folding cofactors and their role in microtubule dynamics. J. Struct. Biol. 135, 219–229 (2001). 11580271 10.1006/jsbi.2001.4386

[b3] Tian G. . Pathway leading to correctly folded beta-tubulin. Cell 86, 287–296 (1996). 8706133 10.1016/s0092-8674(00)80100-2

[b4] Stomacher M., Goldman R. D., Louvard D. & Vignjevic D. M. Actin, microtubules, and vimentin intermediate filaments cooperate for elongation of invadopodia. J. Cell Biol. 189, 541–556 (2010). 20421424 10.1083/jcb.200909113 PMC2867303

[b5] Lewis S. A., Tian G. & Cowan N. The α- and β-tubulin folding pathways. Trends Cell Biol. 7, 479–484 (1997). 17709011 10.1016/S0962-8924(97)01168-9

[b6] Lytle B. L. . Solution structure of a ubiquitin-like domain from tubulin-binding cofactor B. J. Biol. Chem. 279, 46787–46793 (2004). 15364906 10.1074/jbc.M409422200

[b7] Wang W. . Gigaxonin interacts with tubulin folding cofactor B and controls its degradation through the ubiquitin-proteasome pathway. Curr. Biol. 15, 2050–2055 (2005). 16303566 10.1016/j.cub.2005.10.052

[b8] Lopez-Fanarraga M. . Tubulin cofactor B plays a role in the neuronal growth cone. J. Neurochem. 100, 1680–1687 (2007). 17217416 10.1111/j.1471-4159.2006.04328.x

[b9] Tian G. . A Pachygyria-causing alpha-tubulin mutation results in inefficient cycling with cct and a deficient interaction with TBCB. Mol. Biol. Cell 19, 1152–1161 (2008). 18199681 10.1091/mbc.E07-09-0861 PMC2262973

[b10] Vadlamudi R. K. . p21-activated kinase 1 regulates microtubule dynamics by phosphorylating tubulin cofactor B. MCB. 25, 3726–3736 (2005). 15831477 10.1128/MCB.25.9.3726-3736.2005 PMC1084301

[b11] Rayala S. K. . Dynamic interplay between nitration and phosphorylation of tubulin cofactor B in the control of microtubule dynamics. Pnas. 104, 19470–19475 (2007). 18048340 10.1073/pnas.0705149104 PMC2148313

[b12] Tian G. . Tubulin subunits exist in an activated conformational state generated and maintained by protein cofactors. J. Cell. Biol. 138, 821–832 (1997). 9265649 10.1083/jcb.138.4.821 PMC2138046

[b13] Tian G. . Disease-associated mutations in TUBA1A result in a spectrum of defects in the tubulin folding and heterodimer assembly pathway. Hum. Mol. Genet. 19, 3599–3613 (2010). 20603323 10.1093/hmg/ddq276 PMC2928131

[b14] Carranza G. . Auto inhibition of TBCB regulates EB1-mediated microtubule dynamics. Cell Mol. Life Sci. 70, 357–371 (2013). 22940919 10.1007/s00018-012-1114-2 PMC11113326

[b15] Garcia de la serrana D. & Johnston I. A. Expression of Heat Shock Protein (Hsp90) Paralogues Is Regulated by Amino Acids in Skeletal Muscle of Atlantic Salmon. PLoS One 8, e74295 (2013). 24040223 10.1371/journal.pone.0074295 PMC3765391

[b16] Cox D. N. . A novel class of evolutionarily conserved genes defined by piwi are essential for stem cell self-renewal. Genes Dev. 12, 3715–3727 (1998). 9851978 10.1101/gad.12.23.3715 PMC317255

[b17] Qiao D., Zeeman A. M., Deng W., Looijenga L. H. & Lin H. Molecular characterization of hiwi, a human member of the piwi gene family whose overexpression is correlated to seminomas. Oncogene 21, 3988–3999 (2002). 12037681 10.1038/sj.onc.1205505

[b18] Suzuki R., Honda S. & Kirino Y. PIWI expression and function in cancer. Frontiers in Genetics http://dx, doi: 10.3389/fgene.2012. 00204 (2012). PMC3472457 23087701

[b19] Cerutti L., Mian N. & Bateman A. Domains in gene silencing and cell differentiation proteins: the novel PAZ domain and redefinition of the Piwi domain. Trends Biochem. Sci. 25, 481–482 (2000). 11050429 10.1016/s0968-0004(00)01641-8

[b20] Aravin A. A., Hannon G. J. & Brennecke J. The Piwi-piRNA pathway provides an adaptive defense in the transposon arms race. Science 318, 761–764 (2007). 17975059 10.1126/science.1146484

[b21] Sasaki T., Shiohama A., Minoshima S. & Shimizu N. Identification of eight members of the Argonaute family in the human genome small star, filled. Genomics 82, 323–330 (2003). 12906857 10.1016/s0888-7543(03)00129-0

[b22] Lee J. H. . Stem-cell protein Piwil2 is widely expressed in tumors and inhibits apoptosis through activation of Stat3/Bcl-XL pathway. Hum. Mol. Genet. 15, 201–211 (2006). 16377660 10.1093/hmg/ddi430

[b23] Seto A. G., Kingston R. E. & Lau N. C. The coming of age for Piwi proteins. Mol. Cell 26, 603–609 (2007). 17560367 10.1016/j.molcel.2007.05.021

[b24] Liu J. J. . Piwil2 is expressed in various stages of breast cancers and has the potential to be used as a novel biomarker. Int. J. Clin. Exp. Pathol. 3, 328–337 (2010). 20490325 PMC2872741

[b25] He G. . Piwil2 expressed in various stages of cervical neoplasia is a potential complementary marker for p16. Am. J. Transl. Res. 2, 156–169 (2010). 20407605 PMC2855633

[b26] Ye Y. . Identification of Piwil2-like (PL2L) proteins that promote tumorigenesis. PLoS One 5, e13406 (2010). 20975993 10.1371/journal.pone.0013406 PMC2958115

[b27] Lu Y. . Identification of piRNAs in HeLa cells by massive parallel sequencing. BMB. Rep. 43, 635–641 (2010). 20846497 10.5483/BMBRep.2010.43.9.635

[b28] Zhang K. . HILI Inhibits TGF-β Signaling by Interacting with Hsp90 and Promoting TβR Degradation. PLoS One 7, e41973 (2012). 22848678 10.1371/journal.pone.0041973 PMC3407066

[b29] Yao Y. . PIWIL2 induces c-Myc expression by interacting with NME2 and regulates c-Myc-mediated tumor cell proliferation. Oncotarget 5, 8466–8477 (2014). 25193865 10.18632/oncotarget.2327 PMC4226697

[b30] Lee J. H. . Pathways of Proliferation and Antiapoptosis Driven in Breast Cancer Stem Cells by Stem Cell Protein Piwil2. Cancer Res. 70, 4569–4579 (2010). 20460541 10.1158/0008-5472.CAN-09-2670

[b31] Yin D. . Germline stem cell gene PIWIL2 mediates DNA repair through relaxation of chromatin. PLoS One 6, e27154 (2011). 22110608 10.1371/journal.pone.0027154 PMC3217960

[b32] Jiang S. . Piwil2 Inhibits Keratin 8 Degradation through Promoting p38-Induced Phosphorylation To Resist Fas-Mediated Apoptosis. MCB. 34, 3928–3938 (2014). 25113562 10.1128/MCB.00745-14 PMC4386451

[b33] Schulze E., Asai D. J., Bulinski J. C. & Kirschner M. Posttranslational modification and microtubule stability. J. Cell Biol. 105, 2167–2177 (1987). 3316248 10.1083/jcb.105.5.2167 PMC2114866

[b34] Webster D. R. & Borisy G. G. Microtubules are acetylated in domains that turn over slowly. J. Cell Sci. 92, 57–65 (1989). 2674164 10.1242/jcs.92.1.57

[b35] Fanarraga M. L., Villegas J. C., Carranza G., Castaño R. & Zabala J. C. Tubulin cofactor B regulates microtubule densities during microglia transition to the reactive states. Exp. Cell Res. 315, 535–541 (2009). 19038251 10.1016/j.yexcr.2008.10.045

[b36] Espindola F. S. . The light chain composition of chicken brain myosin—Va: calmodulin, myosin—II essential light chains, and 8-kDa dynein light chain/PIN. Cell Motil. Cytoskeleton 47, 269–281 (2000). 11093248 10.1002/1097-0169(200012)47:4<269::AID-CM2>3.0.CO;2-G

[b37] White S. R. . Initiation of Apoptosis by Actin Cytoskeletal Derangement in Human Airway Epithelial cells. Am. J. Respir. Cell Mol. Biol. 24, 282–294 (2001). 11245627 10.1165/ajrcmb.24.3.3995

[b38] Mykkänen O. M. . Characterizationof human paladin, a microfilament- associated protein. Mol. Biol. Cell 2, 3060–3073 (2001). 10.1091/mbc.12.10.3060 PMC60155 11598191

[b39] Kerssemakers J. W. . Assembly dynamics of microtubules at molecular resolution. Nature 442, 709–712 (2006). 16799566 10.1038/nature04928

[b40] Vasiliev J. M. & Gelfand I. M. Morphogenetic reactions and locomotory behavior of transformed cells in culture. In Fundamental Aspects of Metastasis ed. Weiss L., Amsterdam, NorthHolland, 71–98 (1976).

[b41] Bershadsky A. D., Vaisberg E. A. & Vasiliev J. M. Pseudopodial activity at the active edge of migrating fibroblast is decreased after drug-induced microtubule depolymerization. Cell Motil. Cytoskeleton 19, 152–158 (1991). 1878985 10.1002/cm.970190303

[b42] Bershadsky A., Chausovsky A., Becker E., Lyubimova A. & Geiger B. Involvement of microtubules in the control of adhesion-dependent signal transduction. Curr. Biol. 6, 1279–1289 (1996). 8939572 10.1016/s0960-9822(02)70714-8

[b43] Enomoto T. Microtubule disruption induces the formation of actin stress fibers and focal adhesions in cultured cells: possible involvement of the rho signal cascade. Cell Struct. Funct. 21, 317–326 (1996). 9118237 10.1247/csf.21.317

